# Drug Use Patterns in Myasthenia Gravis: A Real-World Population-Based Cohort Study in Italy

**DOI:** 10.3390/jcm13113312

**Published:** 2024-06-04

**Authors:** Marco Finocchietti, Giada Crescioli, Olga Paoletti, Paola Brunori, Francesco Sciancalepore, Marco Tuccori, Antonio Addis, Alfredo Vannacci, Niccolò Lombardi, Ursula Kirchmayer, The CAESAR Study Group

**Affiliations:** 1 Department of Epidemiology, Lazio Regional Health Service, 00147 Rome, Italy; m.finocchietti@deplazio.it (M.F.); a.addis@deplazio.it (A.A.); u.kirchmayer@deplazio.it (U.K.); 2 Section of Pharmacology and Toxicology, Department of Neurosciences, Psychology, Drug Research and Child Health, University of Florence, 50122 Florence, Italy; giada.crescioli@unifi.it (G.C.); alfredo.vannacci@unifi.it (A.V.); 3 Tuscan Regional Centre of Pharmacovigilance, 50139 Florence, Italy; m.tuccori@ao-pisa.toscana.it; 4 Pharmacoepidemiology Unit, Regional Health Agency of Tuscany, 50141 Florence, Italy; olga.paoletti@ars.toscana.it; 5 Neurophysiopathology, Perugia Hospital, 06129 Perugia, Italy; paola.brunori@ospedale.perugia.it; 6 National Center for Disease Prevention and Health Promotion, Italian National Institute of Health, 00161 Rome, Italy; francesco.sciancalepore@guest.iss.it; 7 Unit of Pharmacology and Pharmacovigilance, Department of Clinical and Experimental Medicine, University of Pisa, 56126 Pisa, Italy

**Keywords:** cohort study, corticosteroid, drug use, myasthenia gravis, pharmacoepidemiology, pyridostigmine

## Abstract

**Background**: In the context of a comparative study of efficacy and safety of drugs used in rare neuromuscular and neurodegenerative diseases (CAESAR—call AIFA_FV_2012-13-14), we assessed the use patterns of drugs indicated for myasthenia gravis (MG). **Methods**: A retrospective cohort study was conducted based on administrative healthcare data. For a cohort of MG patients, prevalent and incident use of pyridostigmine (Py) and other indicated drugs in the first year after case identification was evaluated. Prevalent combined use of major therapies (azathioprine (Az), prednisone (Pr), vitamin D (Vd)) stratified by Py use was assessed, and a comparison between therapies at the time of MG identification and during the first year of follow-up was performed. **Results**: We included 2369 MG patients between 2013 and 2019. Among them, prevalent and incident Py users were 38.4% and 22.0%, respectively. In the first year of follow-up, the use of Pr was observed in 74.5% of Py prevalent users and in 82.0% of Py incident users, respectively; the use of Az was observed in 24.9% and 23.0%, respectively; and the use of Vd was observed in 53.3% and 48.2%, respectively. Among 910 Py prevalent users, 13.1% also used Az, Pr, and Vd, while 15.3% used none of these. Among 938 non-Py users, 2.7% used Az, Pr, and Vd, while 53.8% used none of these. During the first year, an increase in combined therapies was evident in incident Py users. **Conclusions**: Our results suggest that, for some MG patients, there may be a need for treatments that combine a rapid onset of benefit with long-term and consistent disease control. These issues may be addressed by the new treatments currently being developed. To date, more studies are needed to address the heterogeneity, quality, and generalizability of the existing data and to evaluate patterns of use, efficacy, and safety of new or emerging therapies for MG.

## 1. Introduction

Myasthenia gravis (MG) is a chronic autoimmune disorder that affects neuromuscular transmission, resulting in fluctuating and fatigable muscle weakness [1,2]. The main pathogenic mechanism is the production of autoantibodies that target proteins involved in the formation and function of the neuromuscular junction, such as the acetylcholine receptor (AChR), the muscle-specific kinase (MuSK), the low-density lipoprotein receptor-related protein 4 (LRP4), and agrin [3]. These autoantibodies impair synaptic transmission by reducing the number, density, or function of AChRs or by disrupting the clustering and stabilisation of AChRs in the postsynaptic membrane.

MG can be classified into different subgroups according to the clinical features, the age of onset, the presence and type of autoantibodies, and the thymus pathology [4]. The clinical manifestations vary from isolated ocular symptoms to generalised weakness involving bulbar, respiratory, and limb muscles [5]. The diagnosis of MG is based on the clinical history, physical examination, neurophysiological tests, pharmacological tests, and serological tests [6].

The treatment of MG aims to improve the symptoms, prevent exacerbations, and induce remission [5,7]. The therapeutic options include symptomatic treatment with acetylcholinesterase inhibitors (AChE-Is), immunosuppressive drugs, and intravenous immunoglobulin (IVIg) [8,9]. In particular, anticholinesterase inhibitors, such as pyridostigmine, are the first-line drugs for MG [10]. They enhance the availability of acetylcholine at the neuromuscular junction by inhibiting its degradation by acetylcholinesterase. They are effective for mild to moderate MG, especially for ocular and bulbar symptoms. However, they have limited efficacy for severe or generalised MG and may cause side effects such as abdominal cramps, diarrhoea, excessive salivation, or cholinergic crisis [11]. Immunosuppressive agents, such as corticosteroids, azathioprine, mycophenolate mofetil, cyclosporine, tacrolimus, or methotrexate, are used for moderate to severe MG or for patients who are refractory to anticholinesterase inhibitors [11]. They reduce the production of autoantibodies and modulate the immune system. They are usually initiated at low doses and gradually increased until clinical improvement is achieved or adverse effects occur. They may take several weeks or months to show their full effect and require long-term monitoring for potential complications such as infections, diabetes, osteoporosis, or malignancies. Immunomodulatory therapies, such as intravenous immunoglobulin (IVIg) [12] or plasma exchange (PLEX) [13,14], are used for acute exacerbations of MG or for patients who are unresponsive to other treatments [15]. They act by removing or neutralising circulating autoantibodies and inflammatory mediators. They have a rapid onset of action but a short duration of effect. They are usually reserved for short-term use due to their high cost and risk of adverse reactions, such as allergic reactions, thromboembolic events, or infections. Thymectomy and supportive care have also proved beneficial. The choice of treatment for MG depends on several factors, such as the type and severity of symptoms, the presence of autoantibodies or thymoma, the patient’s age and comorbidities, the availability and cost of drugs, and the patient’s preferences and adherence [16]. The treatment should be individualised and adjusted according to the clinical response and tolerability. The goal of treatment is to achieve minimal manifestations or better status with minimal side effects.

Limited knowledge exists of treatment patterns in clinical practice in patients with MG. A recent study by Mahic and colleagues reported that several MG patients in the United States experienced exacerbations and received rescue therapy despite treatment [17]. Another nationwide drug utilisation study conducted in Denmark found that treatment of MG corresponded to the expected clinical course of the disease and that most patients underwent long-term immunosuppression [18].

In Italy, evidence regarding the patterns of use of medications in subjects diagnosed with MG is still lacking. Thus, the aim of the present study was to describe the pattern of use of MG pharmacological treatments in clinical practice, analysing administrative data of three Italian regions, taking advantage of the CAESAR project, a multiregional Italian pharmacovigilance project on the comparative effectiveness and safety of drugs used in rare neuromuscular and neurodegenerative diseases.

## 2. Materials and Methods

### 2.1. Study Design

This is a real-world population-based retrospective cohort study, part of a multiregional Italian pharmacovigilance project on the comparative effectiveness and safety of drugs used in rare neuromuscular and neurodegenerative diseases (the CAESAR project) [19,20]. In the period 2013–2019, three Italian regions were involved: Latium, Tuscany, and Umbria.

### 2.2. Data Sources

The following data sources were considered: (1) healthcare assistance (demographic and residence information); (2) hospital discharge records (all hospitalisation information, including principal and secondary diagnoses and procedures, coded according to the ICD-9 cm classification); (3) emergency department (ED) visits (all information regarding ED accesses, including principal and secondary diagnoses coded according to the ICD-9 cm classification, severity of patient conditions and triage parameters); (4) disease-specific co-payment exemptions (exemptions from healthcare service co-payment database, that contains coded information about chronic diseases); (5) drug claims for reimbursed drugs for outpatient use (coded according to the international Anatomical Therapeutic Chemical Classification System (ATC), claim date, quantity of active agent issued); and (6) mortality information system (information about mortality, including date, place of death).

### 2.3. Cohort Selection

In order to perform the analysis, subjects were identified as affected by MG if they met any of the following three criteria in the years 2013–2019: (a) discharge from hospital with a primary diagnosis of MG (ICD-9 cm code 358.0), or a secondary diagnosis in combination with discharge from a hospital neurology ward; (b) discharge from ED with a primary diagnosis of MG (ICD-9 cm code 358.0); (c) disease-specific co-payment exemption for MG (Italian code: RFG101).

The first of these dates was defined as the index date. Patients younger than 18 years, not resident, or not enrolled in the regional healthcare assistance database at the index date and in the two years before the index date were excluded. The two years prior to the index date were defined as the look-back period and the 12 months after the index date as the follow-up period. Drugs of interest in this study were pyridostigmine and other drugs typically indicated in MG, with a focus on prednisone, azathioprine and vitamin D. We defined the use of these drugs in terms of at least one drug claim and divided the main cohort into three cohorts with different patterns of pyridostigmine use as follows: pyridostigmine non-users, pyridostigmine prevalent users (with at least one pyridostigmine dispensation in the look-back and follow-up periods), and pyridostigmine incident users (with at least one pyridostigmine dispensation in the follow-up and no dispensation during look-back). The use of all other drugs was described, stratifying for these three cohorts.

### 2.4. Statistical Analysis

First, the cohorts were characterised at baseline, retrieving information from the look-back period. Frequency distributions were computed for demographical characteristics, MG-specific comorbidities, complications and non-pharmacological therapies (2-year look-back) and pharmacological therapies (1-year look-back). Second, the frequency of drug use during follow-up was described. Then, with respect to our focus, use patterns of prednisone, azathioprine and vitamin D, alone or in combination with pyridostigmine during follow-up, was represented through Venn diagrams. Finally, changes in the use patterns of the above cited therapies between baseline and follow-up were depicted in Sankey diagrams, separately for the three pyridostigmine user cohorts (non-users, prevalent users and incident users). Each Region extracted their own data and performed analysis at the regional level. All databases were linked through an anonymous unique subject identification code. Aggregated data were combined across regions. The full list of ICD-9 cm codes and ATC codes are available in the Supplementary Materials (Tables S1 and S2, respectively).

## 3. Results

**Figure 1:**
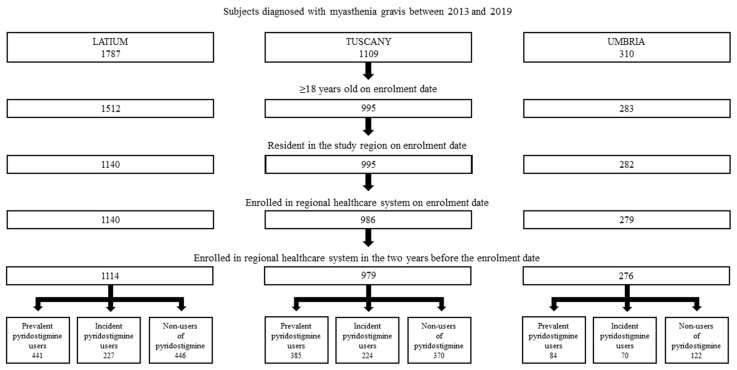
Cohort selection of MG patients in three Italian regions in the period 2013–2019.

Overall, 2369 MG individuals were identified in the three regions in the period 2013–2019 (Figure 1). Of these, 910 were pyridostigmine prevalent users, 521 incident users and 938 non-users, respectively.

Patients’ characteristics at baseline are summarised in Table 1: the percentage of males was 49.9%, 57.8%, and 43.6% in the three groups, respectively. The proportions of patients aged 50 years or older varied between 73.2% in pyridostigmine non-users and 84.3% in pyridostigmine incident users. Main comorbidities and complications in the two years before the index date in the prevalent, incident and non-user groups were myasthenia gravis with (acute) exacerbation (32.9%, 53.4% and 38.3%, respectively), diseases of the circulatory system (25.5%, 42.2% and 25.8%), diseases of the respiratory system (18.1%, 14.0% and 13.3%), and neoplasms (11.3%, 14.0% and 9.8%). Other comorbidities and complications present in about 10% of patients were acute respiratory failure, diabetes mellitus with or without mention of complication, fractures, mental disorders, and autoimmune diseases. With respect to pharmacological therapies at baseline, the most frequent drugs in the pyridostigmine prevalent, incident and non-user cohorts were corticosteroids for systemic use (69.8%, 32.4% and 44.5%), and in particular prednisone (62.7%, 13.6% and 31.1%), other immunosuppressants (15.8%, 1.7% and 5.4%), and in particular azathioprine (15.1%, 1.3% and 4.8%), vitamin D (38.8%, 17.1% and 30.6%), and folic acid (9.5%, 7.7% and 8.0%). Frequency distributions of non-pharmacological therapies showed poor use for the three cohorts. Only mechanical ventilation (3.3%, 3.6% and 1.6%) and plasmapheresis (5.2%, 2.7% and 1.5%) were retrieved in more than 1% of patients in the three cohorts.

**Figure 2:**
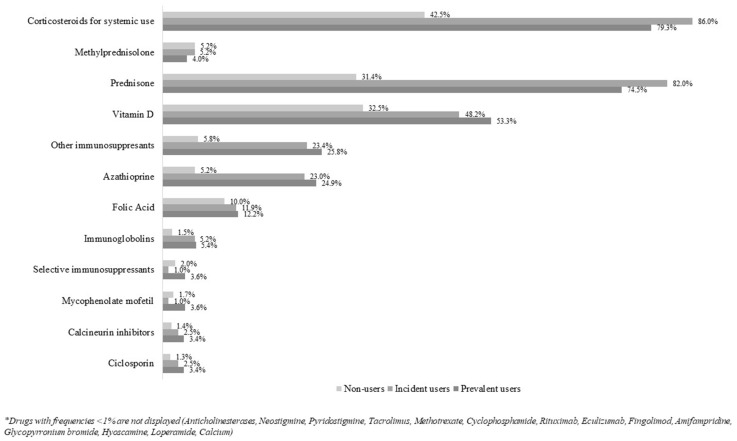
Frequency of drugs indicated for MG during follow-up.

In the year of follow-up (Figure 2 and Figure S1), 79.3% of prevalent pyridostigmine users, 86.0% of incident users and 42.5% of non-users received corticosteroids for systemic use, in particular prednisone (74.5%, 82.0% and 31.4%), other immunosuppressants (25.8%, 23.4% and 5.8%), in particular azathioprine (24.9%, 23.0% and 5.2%), vitamin D (53.3%, 48.2% and 32.5%), and folic acid (12.2%, 11.9% and 10.0%).

**Figure 3:**
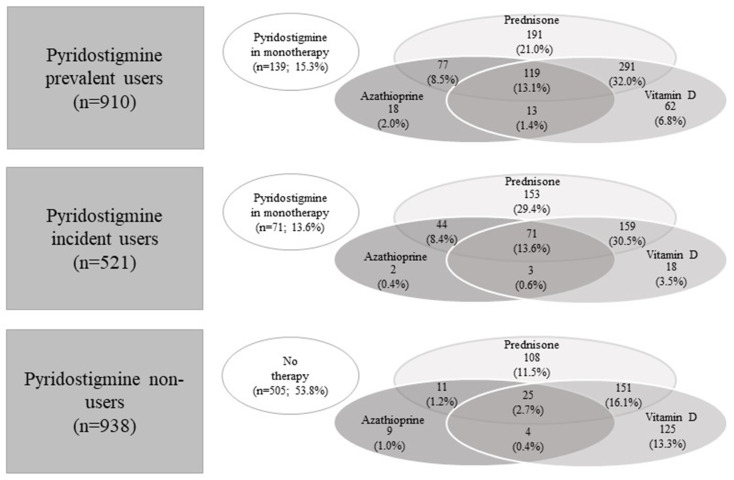
Use patterns of the study drugs during follow-up stratified by pyridostigmine use.

Figure 3 shows the use patterns of the drugs during follow-up for the three pyridostigmine user cohorts. For prevalent pyridostigmine users, the most frequent combinations were pyridostigmine with prednisone and vitamin D (32.0%), pyridostigmine with prednisone (21.0%), pyridostigmine with prednisone, vitamin D and azathioprine (13.1%), while 15.3% used pyridostigmine in monotherapy. In incident pyridostigmine users, 30.5% used pyridostigmine in combination with prednisone and vitamin D, 29.4% in combination with prednisone alone, 13.6% in combination with azathioprine, prednisone, and vitamin D, whereas 13.6% used pyridostigmine in monotherapy. Among non-users of pyridostigmine, 53.8% did not use any of the other drugs, 16.1% used prednisone in combination with vitamin D, 13.3% vitamin D in monotherapy, and 11.5% prednisone in monotherapy.

**Figure 4:**
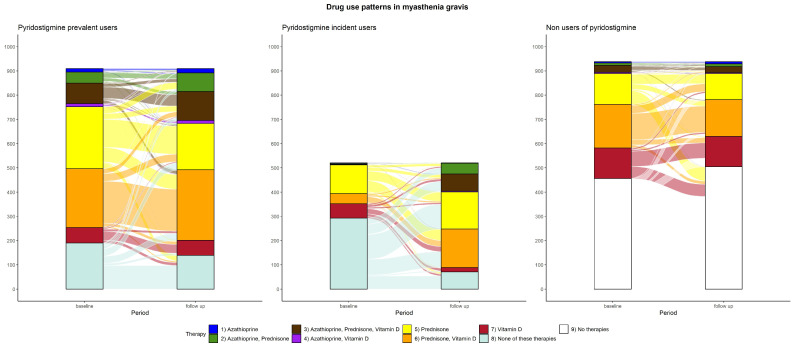
Changes in drug use patterns between baseline and follow-up stratified by pyridostigmine use.

Changes in drug use patterns of the study drugs during the first year are shown in Figure 4, representing the proportions of mono- or combined therapies at baseline and during follow-up. For the cohort of prevalent users, the most frequent patterns at baseline were pyridostigmine with prednisone (28.0%), pyridostigmine with prednisone and vitamin D (26.7%), and pyridostigmine in monotherapy (20.9%). During follow-up, pyridostigmine monotherapy combined with prednisone decreased to 15.3% and 21.0%, respectively, whereas the combination of pyridostigmine with prednisone and vitamin D increased to 32.0%. Overall, combinations of pyridostigmine with two or more indicated drugs increased during follow-up. Among incident users, 56.2% did not use any of the other drugs, 22.7% combined with prednisone, and 11.3% with vitamin D. During follow-up, a general uptake of pyridostigmine was observed, with pyridostigmine monotherapy (13.6%), prednisone from 22.7% to 29.4%, in combination with pyridostigmine. The combination of prednisone, azathioprine and vitamin D increased from 0.4% to 13.6% in combination with pyridostigmine. The combination of azathioprine, prednisone and pyridostigmine in the follow-up related to 8.5% of patients, while at baseline, the therapy based on azathioprine and prednisone was present in only 0.6% of the cohort. The combination of prednisone and vitamin D multiplied from 8.1% to 30.5%, in combination with pyridostigmine. The use of vitamin D decreased from 11.3% to 3.5% in combination with pyridostigmine. For the non-user cohort, the most relevant changes were evident for patients not using any of the drugs, which increased from 48.6% to 53.8%. The combination of prednisone and vitamin D varied from 19.2% to 16.1%, vitamin D monotherapy was stable, and prednisone in monotherapy decreased from 13.5% to 11.5%. The non-user cohort presented stable percentages over time.

## 4. Discussion

The present study investigated the use patterns of drugs indicated in MG in a real-world setting, referring to around 10 million residents. To the best of our knowledge, this is the first drug utilisation study of its kind performed in Italy.

The majority of patients diagnosed with MG are treated with pyridostigmine alone or in combination with prednisone, azathioprine and vitamin D. Corticosteroid for systemic use therapy is more frequent in new users of pyridostigmine. During the observation period, the use of corticosteroids and immunosuppressants increased in both prevalent and incident pyridostigmine users. This is in line with clinical guidelines [21], which recently reported new recommendations for the use of rituximab, eculizumab, and methotrexate and for the early immunosuppression in ocular MG and MG associated with immune checkpoint inhibitor treatment [22]. Data regarding the use of biological products in our sample is relatively low, as highlighted by the frequency of use of drugs such as rituximab and eculizumab amounting to less than 1%. This cannot be directly attributed to the costs of the drugs but rather partly to the study period (2013–2019), during which some of the therapies currently in use were not yet approved in Italy (i.e., efgartigimod, zilucoplan) and partly to the fact that innovative drugs, such as eculizumab or FcRn inhibitors are often administered in a hospital setting first. Drug claims data available in the regional register refer to outpatient claims, whereas hospital therapies are not retrievable. We might, therefore, have missed or underestimated these therapies in our dataset.

MG can have a significant impact on the quality of life and functional status of patients and requires lifelong treatment with immunosuppressive agents, symptomatic drugs, or both [23]. However, there is limited evidence on the optimal pharmacological management of MG, especially for individuals who are refractory to conventional therapies or have comorbidities. Therefore, drug utilisation research in real world settings can help to fill the knowledge gaps and inform clinical decision-making for MG patients. However, few population-based studies of drug use in MG have been published in the scientific literature. In fact, most of the studies in the literature focus on the clinical characteristics of the disease rather than its pharmacological treatments.

In Europe, some studies have reported an analysis comparable to our investigation. A retrospective longitudinal cohort study of 1149 MG patients in England found that patients with refractory MG who did not respond to conventional treatment had more exacerbations and hospitalisations than patients with non-refractory MG. This study also reported that refractory MG patients had higher rates of comorbidities, such as renal disease and hypertension, than non-refractory MG patients and matched controls without MG [24]. Considering the pharmacological treatment reported by Harris et al. [24], during the full follow-up period, patients received a median of two different treatments for MG. According to our results, the most frequently prescribed were pyridostigmine (70.3%), prednisolone (61.6%), and azathioprine (24.9%). IVIg was administered to 9.6% of patients, while plasmapheresis to 2.1% of patients.

Both rituximab and eculizumab may be considered therapeutic options for MG after insufficient symptom control by standard immunosuppressive therapies [23]. A retrospective observational study included 57 rituximab-treated and 20 eculizumab-treated patients with MG to compare their efficacy with an observation period of 24 months [25]. In this study conducted in Germany, eculizumab was associated with a better outcome compared with rituximab, as measured by the change in the quantitative myasthenia gravis score at 12 and 24 months of treatment. However, the risk of the myasthenic crisis was not ameliorated in either group. This study also reported that rituximab was well tolerated and reduced the use of other immunosuppressive drugs. Considering our data, a total of seven individuals were treated with rituximab (five in the group of prevalent users of pyridostigmine and two in the group of non-users) and none with eculizumab.

Another retrospective cohort study estimated the prevalence and incidence of MG in Germany, evaluating the burden of disease and treatment patterns based on anonymised German claims data [26]. Methodologically, this study offers many similarities to our real-world analysis. In fact, Mevius et al. [26] identified two patient samples (incident and prevalent MG patients), analysing and describing a total of 775 incidents (mean age of 66.9 years) and 1247 prevalent MG patients (mean age of 68.6 years). Among all incident MG patients, 31.5% received no MG treatment during the first 12 months after the index diagnosis, while pyridostigmine (60.4%), oral corticosteroids (39.4%), and azathioprine (26.8%) were the most frequently prescribed treatments. Furthermore, thymectomy was observed in 4.4% of the incident patients, and the administration of IVIg in 9.3% of them. Similarly, in our cohorts, 4.3% of thymectomy and 6.3% of IVIg administration were observed.

Overseas, an observational study performed in the United States identified adult patients newly diagnosed with MG from the IBM MarketScan insurance claims database, with the aim of describing MG treatment patterns [17]. During the study period (2010–2019), a total of 7194 patients were followed for up to 10 years. Of 6539 treated patients, 99% were ever treated with AChE-I and/or corticosteroids; 95% were first treated with AChE-I and/or corticosteroids only; 33% received ≥1 non-steroid immunosuppressive treatment and 2% received a biologic. The authors also reported that many patients experienced exacerbations and received rescue therapy despite MG treatment, suggesting current treatments may not provide adequate disease control for some patients and that additional treatment options should be explored. As suggested by the results of the study by Mahic and colleagues [17], the use of drugs indicated for MG is in line with international guidelines [21,22]. In fact, also in America their pattern of use is comparable with that observed in Italy.

Another drug utilisation study performed in the United States evaluated the real-world utilisation patterns of IVIg among patients with generalised MG over 3 years [27]. Patients with generalised MG who initiated IVIg treatment were identified from Symphony Health’s Integrated Dataverse^®^. During the study period (2014–2019), among 1225 patients with generalised MG who initiated IVIg treatment, 57.6% received 1 to 5 IVIg treatment courses (intermittent IVIg users), and 42.4% received ≥6 IVIg treatment courses (chronic IVIg users) within the subsequent year. Of note, the proportion of patients using corticosteroids and nonsteroidal immunosuppressive treatments was not reduced over the 3-year follow-up period following IVIg initiation, even for patients who continued annual chronic IVIg for 3 consecutive years post-initiation. Although IVIg utilisation at the baseline is relatively low in our sample, both in prevalent pyridostigmine users and non-users, we observed a reduction in corticosteroid use, particularly prednisone, during follow-up. This evidence could be explained by the desire to avoid toxicity deriving from chronic use of corticosteroids [28], reducing the risk of cardiovascular events, diabetes and osteoporosis in MG individuals. In this regard, our study indicates that the prescription of vitamin D in individuals with MG may serve dual purposes. Firstly, it could potentially mitigate adverse effects resulting from prolonged corticosteroid therapy. Secondly, it may offer benefits such as fatigue reduction and symptom remission. Existing research suggests that restoring vitamin D levels, especially in MG patients with plasma levels below 30 ng/mL, might contribute to alleviating the proinflammatory milieu associated with autoimmune disorders and potentially impacting disease progression. Noteworthy, studies have reported enhancements in MG-related fatigue with vitamin D supplementation, alongside instances of remission in refractory MG cases following high-dose vitamin D therapy [29,30].

This is even more relevant considering that MG is generally diagnosed at a young age [31] and that the indicated therapies are used throughout life. A systematic review and meta-analysis of the global prevalence of MG and the effectiveness of common drugs in its treatment found that the prevalence of MG worldwide was estimated to be 12.4 people per 100,000 population and that mycophenolate and IVIg or plasma exchange were administered to the majority of MG patients with positive effects in reducing the symptoms and biomarkers of MG [32]. Regarding the usefulness of oral steroids, the authors reported that it is determined by the occurrence of a wide range of dose and time-dependent side effects, which are generally less frequently reported when corticosteroids are administered intravenously.

The present findings have been generated in the context of a multiregional Italian pharmacovigilance project financed by the Italian Medicines Agency (AIFA) and offer information to healthcare providers and decision makers, which may be useful for audit and feedback activities and to the ends of healthcare management decisions. This is particularly valuable in view of forthcoming innovative and often expensive therapies, e.g., biological drugs. In the context of MG, our findings can be viewed as new, useful and feasible real-world insights into the pharmacological management of MG.

### Limitations

The main limitation is inherent to the nature of our data, which does not provide detailed clinical information, for example, with respect to the onset, duration, severity and subtype of the disease. The choice to stratify the patients based on the use of pyridostigmine rather than their identification in the administrative databases was made based on this study’s objective (to provide a picture of drug use in MG in clinical practice in Italy) and also based on sample size. Although patients identified from various administrative databases may have potentially different clinical characteristics, the adjustment of the analyses reduced the influence of covariates on results even in the stratification as it was conducted (based on pyridostigmine use). Future studies with a larger sample size and a longer follow-up may allow us to overcome this issue. In fact, focusing on the drug use patterns linked to enrolment identification might enhance the understanding of pharmacological treatments in MG. Moreover, it is to be taken into account that our enrolment date may not be the true start of the disease. Of notice, these limitations may have different impacts in the three regions, because traceability of drug claims depends on the administrative procedures underlying drug registration under different reimbursement policies [19,20,33]. Furthermore, treatments administered to in-patients (immunomodulation therapies such as intravenous immunoglobulins and plasma exchange) and self-prescribed drugs (i.e., over-the-counter medications) are generally not retrievable at the patient level. As a whole, population-based studies can provide useful insights into the management and outcomes of MG patients. Therefore, cohort studies should be interpreted with caution and complemented by other types of studies, such as systematic reviews and meta-analyses. Finally, due to Italian National privacy legislation, we were unable to extend the follow-up period beyond 12 months.

On the other hand, to the best of our knowledge, there are no prior publications on this topic in Italy and very few at the international level. The main strength of our study is the population-based approach and the availability of data referring to the resident population, which includes relevant patient demographic and clinical covariates. Furthermore, the identified cohorts are not selected, and the analysis includes a large sample of individuals affected by MG despite MG being a rare disease. Additionally, even if the information on individual doses is not available in our databases, medications investigated are all reimbursed by the healthcare service and have, therefore, been well evaluated. Finally, this is a multicentre study, providing evidence from different Italian regions that represent approximately 18% of the Italian residents, making these results generalisable to the entire country’s population.

## 5. Conclusions

Our real-world analysis showed that the management of MG patients in clinical practice generally follows guideline suggestions. Unexpectedly, we found a relevant number of patients who were not receiving treatment within 12 months from diagnosis. Despite available treatment options, the impact of the disease remains extremely high for many MG patients, especially those with higher clinical burdens and concomitant diseases. Our results emphasise that, for MG patients, there remains an unmet need for treatments that combine a rapid onset of benefit with long-term and consistent disease control. These issues may be addressed by the new treatments currently being developed. To date, more studies are needed to address the heterogeneity, quality, and generalizability of the existing data, and to evaluate patterns of use, efficacy and safety of new or emerging therapies for MG.

## Figures and Tables

**Table 1:** Patients’ characteristics at baseline.

	Prevalent Pyridostigmine Users		Incident Pyridostigmine Users		Pyridostigmine Non-Users	
	910		521		938	
	n	%	n	%	n	%
**Demographical characteristics**						
**Sex**						
Females	456	50.1%	220	42.2%	529	56.4%
Males	454	49.9%	301	57.8%	409	43.6%
**Age**						
18–49	184	20.2%	82	15.7%	251	26.8%
50+	726	79.8%	439	84.3%	687	73.2%
**Complications and comorbidities**						
Acute respiratory failure	53	5.8%	24	4.6%	28	3.0%
Myasthenia gravis with (acute) exacerbation	299	32.9%	278	53.4%	359	38.3%
Benign neoplasm of thymus	6	0.7%	14	2.7%	5	0.5%
Malignant neoplasm of thymus	34	3.7%	5	1.0%	8	0.9%
Diabetes mellitus	68	7.5%	41	7.9%	51	5.4%
Diabetes mellitus without mention of complications	65	7.1%	36	6.9%	42	4.5%
Other osteoporosis	14	1.5%	0	0.0%	7	0.7%
Fractures	49	5.4%	31	6.0%	60	6.4%
Obstructive chronic bronchitis	41	4.5%	9	1.7%	28	3.0%
Overweight, obesity and other hyperalimentation	19	2.1%	11	2.1%	10	1.1%
Neoplasms	103	11.3%	73	14.0%	92	9.8%
Mental disorders	44	4.8%	39	7.5%	66	7.0%
Diseases of the circulatory system	232	25.5%	220	42.2%	242	25.8%
Diseases of the respiratory system	165	18.1%	73	14.0%	125	13.3%
Autoimmune diseases	37	4.1%	25	4.8%	40	4.3%
**Pharmacological therapy**						
Anticholinesterases	897	98.6%	0	0.0%	99	10.6%
Pyridostigmine	897	98.6%	0	0.0%	99	10.6%
Corticosteroids for systemic use	635	69.8%	169	32.4%	417	44.5%
*Methylprednisolone*	61	6.7%	46	8.8%	70	7.5%
*Prednisone*	571	62.7%	71	13.6%	292	31.1%
Selective immunosuppressants	21	2.3%	0	0.0%	13	1.4%
*Mycophenolate mofetil*	21	2.3%	0	0.0%	10	1.1%
Calcineurin inhibitors	26	2.9%	2	0.4%	16	1.7%
*Ciclosporin*	25	2.7%	2	0.4%	15	1.6%
Other immunosuppressants	144	15.8%	9	1.7%	51	5.4%
*Azathioprine*	137	15.1%	7	1.3%	45	4.8%
Immunoglobulins	38	4.2%	4	0.8%	12	1.3%
Vitamin D	353	38.8%	89	17.1%	287	30.6%
Calcium	20	2.2%	3	0.6%	16	1.7%
Folic Acid	86	9.5%	40	7.7%	75	8.0%
**Non-pharmacological therapy**						
Thymectomy	28	3.1%	3	0.6%	6	0.6%
Non-invasive mechanical ventilation	8	0.9%	4	0.8%	0	0.0%
Invasive mechanical ventilation	26	2.9%	16	3.1%	15	1.6%
Mechanical ventilation	30	3.3%	19	3.6%	15	1.6%
Plasmapheresis	47	5.2%	14	2.7%	14	1.5%

* Complications and comorbidities with frequencies <1% are not displayed (other chronic hepatitis, malignant essential hypertension, toxic cataract, Cushing’s syndrome, poisoning with parasympathomimetics, other and unspecified non-infectious gastroenteritis and colitis, other specified cardiac dysrhythmias, other and unspecified hyperlipidaemia, cramp of limb, inflammatory diseases of the central nervous system). ** Drugs with frequencies <1% are not displayed (neostigmine, rituximab, eculizimab, amifampridine, glycopyrronium bromide, hyoscamine, loperamide, Fingolimod, Tacrolimus, Methotrexate, Cyclophosphamide).

## Data Availability

The datasets generated and/or analysed during the current study are not publicly available because of privacy reasons.

## Supplementary Materials

The following supporting information can be downloaded at https://www.mdpi.com/article/10.3390/jcm13113312/s1, Figure S1: Frequency of new use of drugs indicated for MG during follow-up; Table S1: ICD-9 cm codes; Table S2: ATC codes.
