# Supplementary material for: Drug Use Patterns in Myasthenia Gravis: A Real-World Population-Based Cohort Study in Italy
Source: J Clin Med. 2024 Jun 4;13(11):3312. doi: 10.3390/jcm13113312 (PMC11172965; doi:10.3390/jcm13113312)
Supplement: Supplementary file 1 [file jcm-13-03312-s001.zip › jcm-2975181-supplementary.pdf]

Supplementary Material

Supplementary Figure S1. Frequency of new use of drugs indicated for MG during follow-up.

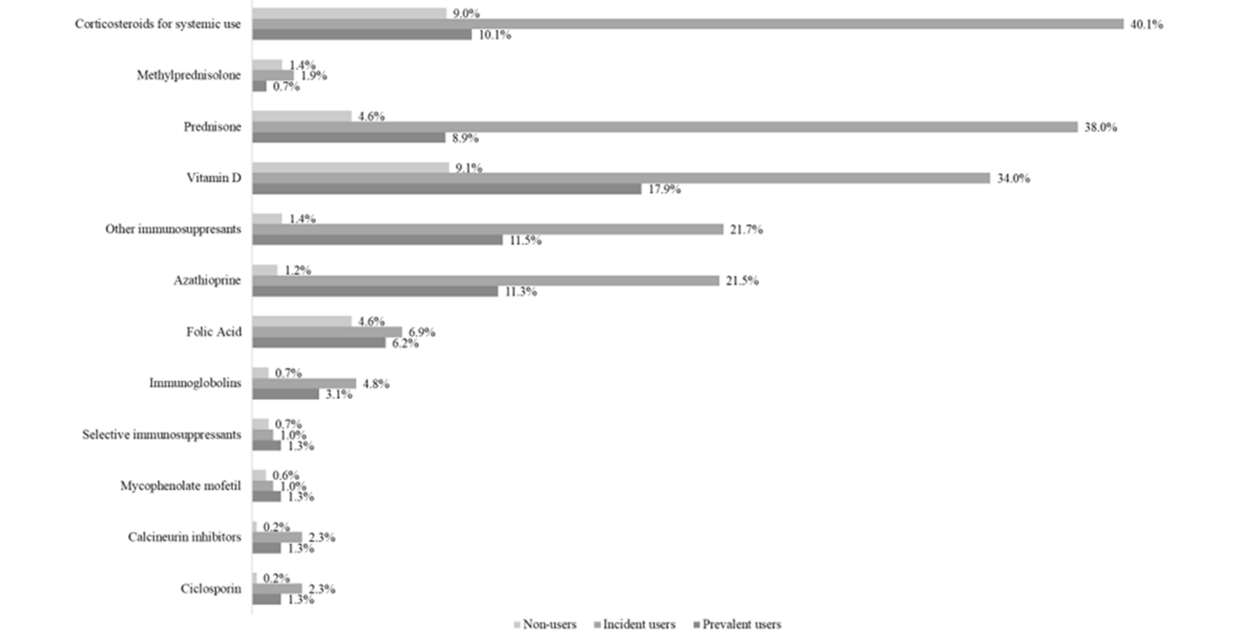

**Drugs with frequencies <1% are not displayed (Anticholinesterases, Neostigmine, Pyridostigmine, Tacrolimus, Methotrexate, Cyclophosphamide, Rituximab, Eculizumab, Fingolimod, Amifampridine, Glycopyrronium bromide, Hyoscamine, Loperamide, Calcium)*

Supplementary Table S1. ICD-9-CM codes.

**Disorder**	**ICD-9-CM diagnosis code**
Myasthenia gravis	358.0
Acute respiratory failure	518.81
Myasthenia gravis with (acute) exacerbation	358.01
Benign neoplasm of thymus	212.6
Malignant neoplasm of thymus	164.0
Thyrotoxicosis	242.9, 242.0
Chronic lymphocytic thyroiditis	245.2
Systemic lupus erythematosus	710.0
Rheumatoid arthritis	714.0
Pernicious anemia	281.0
Other chronic hepatitis	571.49
Psoriasis and similar disorders	696
Malignant essential hypertension	401.0
Diabetes mellitus	250
Diabetes mellitus without mention of complication	250.0
Diabetes with neurological manifestations	250.6
Toxic cataract	366.45
Other osteoporosis	733.09
Fractures	800-829
Cushing's syndrome	255.0
Poisoning with parasympathomimetics [cholinergics]	971.0
Obstructive chronic bronchitis	491.2
Other and unspecified noninfectious gastroenteritis and colitis	558
Other specified cardiac dysrhythmias	427.8
Other and unspecified hyperlipidemia	272.4
Overweight, obesity and other hyperalimentation	278
Cramp of limb	729.82
Neoplasms	140-239
Mental disorders	290-319
Inflammatory diseases of the central nervous system	320-326
Diseases of the circulatory system	390-459
Diseases of the respiratory system	460-519
Autoimmune diseases	135, 136.1, 242, 245.2, 250.01, 250.03, 250.11, 250.13, 250.31, 250.33, 250.41, 250.43, 250.51, 250.53, 250.61, 250.63, 250.71, 250.73, 250.81, 250.83, 250.91, 250.93, 266.2, 281.0, 281.1, 283.0, 287.0, 340, 341.0, 446, 447.6, 555, 556, 694.4, 695.4,

	696, 704.0, 709.01, 710.0, 710.1, 710.2, 710.4, 714.0, 720.0
**Procedure**	**ICD-9-CM procedure code**
Thymectomy	07.8
Non-invasive mechanical ventilation	93.90
Invasive mechanical ventilation	96.7
Plasmapheresis	99.71

Supplementary Table S2. ATC codes.

**Active agent**	**ATC code**
Anticholinesterases	N07AA
Neostigmine	N07AA01
Pyridostigmine	N07AA02
Corticosteroids for systemic use	H02A, H02B
Methylprednisolone	H02AB04
Prednisone	H02AB07
Selective immunosuppressants	L04AA
Mycophenolate mofetil	L04AA06
Fingolimod	L04AA27
Calcineurin inhibitors	L04AD
Ciclosporin	L04AD01
Tacrolimus	L04AD02
Other immunosuppresants	L04AX
Azathioprine	L04AX01
Methotrexate	L04AX03
Cyclophosphamide	L01AA01
Rituximab	L01XC02
Eculizimab	L04AA25
Immunoglobolins	J06B
Amifampridine	N07XX05
Glycopyrronium bromide	A03AB02
Hyoscamine	A03BA03
Loperamide	A07DA05, A07DA03, A07DA53
Vitamin D	A11CC
Calcium	A12AA
Folic Acid	B03BB01
